# Maternal COVID-19 vaccine acceptance among Malaysian pregnant women: A multicenter cross-sectional study

**DOI:** 10.3389/fpubh.2023.1092724

**Published:** 2023-02-22

**Authors:** Aida Kalok, Wira Razak Dali, Shalisah Sharip, Bahiyah Abdullah, Maherah Kamarudin, Rima Anggrena Dasrilsyah, Rahana Abdul Rahman, Ixora Kamisan Atan

**Affiliations:** ^1^Department of Obstetrics and Gynaecology, Faculty of Medicine, National University of Malaysia, Universiti Kebangsaan Malaysia Medical Centre, Kuala Lumpur, Malaysia; ^2^Department of Psychiatry, Faculty of Medicine, National University of Malaysia, Universiti Kebangsaan Malaysia Medical Centre, Kuala Lumpur, Malaysia; ^3^Department of Obstetrics and Gynaecology, Faculty of Medicine, Universiti Teknologi MARA (UiTM), Selangor, Malaysia; ^4^Maternofetal and Embryo (MatE) Research Group, Faculty of Medicine, Universiti Teknologi MARA, Selangor, Malaysia; ^5^Department of Obstetrics and Gynaecology, Faculty of Medicine, Universiti Malaya, Kuala Lumpur, Malaysia; ^6^Department of Obstetrics and Gynaecology, Faculty of Medicine and Health Sciences, Universiti Putra Malaysia (UPM), Selangor, Malaysia

**Keywords:** COVID-19, SARS-CoV-2 vaccine, vaccine acceptance, vaccine hesitancy, pregnancy

## Abstract

**Introduction:**

The coronavirus disease 2019 (COVID-19) caused a global pandemic that resulted in devastating health, economic and social disruption. Pregnant mothers are susceptible to COVID-19 complications due to physiological and immunity changes in pregnancy. We aimed to assess the maternal vaccine acceptance of the COVID-19 vaccine.

**Methods:**

A multi-center study across four teaching hospitals in the Klang Valley, Malaysia was conducted between September 2021 and May 2022. A survey was conducted using a self-administered electronic questionnaire. The survey instruments included; (1) maternal perception and attitude toward COVID-19 vaccination, (2) COVID-19 pregnancy-related anxiety, and 3) generalized anxiety disorder.

**Results:**

The response rate was 96.6%, with a final number for analysis of 1,272. The majority of our women were Malays (89.5%), with a mean age (standard deviation, SD) of 32.2 (4.6). The maternal vaccine acceptance in our study was 77.1%. Household income (*p* < 0.001), employment status (*p* = 0.011), and health sector worker (*p* = 0.001) were independent predictors of maternal willingness to be vaccinated. COVID-19 infection to self or among social contact and greater COVID-19 pregnancy-related anxiety were associated with increased odds of accepting the SARS-CoV-2 vaccine. Women who rely on the internet and social media as a source of vaccine information were more likely to be receptive to vaccination (adjusted odd ratio, AOR 1.63; 95% CI 1.14–2.33). Strong correlations were observed between maternal vaccine acceptance and the positive perception of (1) vaccine information (*p* < 0.001), (2) protective effects of vaccine (*p* < 0.001), and (3) getting vaccinated as a societal responsibility (*p* < 0.001).

**Discussion:**

The high maternal vaccine acceptance rate among urban pregnant women in Malaysia is most likely related to their high socio-economic status. Responsible use of the internet and social media, alongside appropriate counseling by health professionals, is essential in reducing vaccine hesitancy among pregnant women.

## 1. Introduction

The detection of a novel coronavirus in Wuhan, China, at the end of 2019, had led to a global pandemic that caused distressing health, economic and social impacts (1). As of 15 December 2022, over 645 million people have been infected worldwide, with 6.6 million recorded mortalities (2). Malaysia reported around 4.8 million cases, with a death rate of 0.80% (3, 4). The rapid increase in the number of positive cases and deaths during the pandemic; had caused severe strain on the national health system (5). The risk of severe COVID-19 among pregnant women may be higher than in the general population. Physiological and immunity changes during pregnancy increase the women's susceptibility to severe disease (6). Published data have demonstrated a significant association between pregnancy and increased risk of the need for invasive ventilation, ICU admission, and maternal mortalities (7). In addition, Malaysia recorded 191 maternal deaths from COVID-19 complications in 2021 (8).

COVID-19 vaccines were developed to achieve herd immunity and end the current pandemic. Malaysia started its vaccination program on 24th February 2021, and over 72 million vaccines have been administered, and 84.6% of the eligible individuals have received at least two doses of the COVID-19 vaccine (3). Royal College of Obstetricians and Gynecologists has recommended COVID-19 vaccination in pregnancy based on the latest evidence (9). Recent guideline by the Malaysian Ministry of Health has also advised vaccination against COVID-19 among pregnant women between 14 and 33 weeks of gestation (10). Ministry of Health data demonstrated that 79% of pregnant women who died from COVID-19 complications did not receive any vaccination (8).

Vaccine hesitancy is defined as a delay in acceptance or refusal of vaccines despite the availability of vaccine services (11). Vaccine hesitancy can be a hurdle to a successful vaccination program and is a complex behavior subjected to social, cultural, and religious influence (12). The resurgence of vaccine-preventable illnesses has led the WHO to identify vaccine hesitancy as a major threat to global health (13). Beliefs in vaccine effectiveness and safety, fear of side effects, trust in the vaccine's delivery system, and healthcare workers' recommendations are among the factors influencing vaccine acceptance and hesitancy (14, 15). Vaccine hesitancy may be fueled by health information obtained from various sources, including the Internet and social media platforms, that gained global penetrance as the technology improved (13).

An online survey in sixteen countries involving antenatal women and mothers of young children found that 52.0% of pregnant women and 73.4% of non-pregnant women indicated an intention to receive the vaccine. The strongest predictors of vaccine acceptance included confidence in vaccine safety or effectiveness, worry about COVID-19, and trust in public health agencies/health science (16). A French study demonstrated that only one-third of expectant mothers would be willing to be vaccinated (17). Egloff et al. (18) found that the main reason for not agreeing was being more afraid of the potential side effects of the SARS-CoV-2 vaccine on the fetus than of COVID-19. A cross-sectional study from the USA showed that less than half of the women surveyed were willing to get vaccinated during pregnancy (18).

Our study aimed to evaluate the maternal perception and attitude toward COVID-19 vaccination and the factors influencing vaccine hesitancy among expectant mothers in Malaysia.

## 2. Methods

We conducted a multi-center cross-sectional study between September 2021 and May 2022, that involved four teaching hospitals across the Klang Valley in Malaysia; (1) Universiti Kebangsaan Malaysia Medical Center, (2) Universiti Malaya Medical Center, (3) Universiti Teknologi MARA Hospital and (4) Universiti Putra Malaysia Teaching Hospital. This study was a research collaboration between four major public universities, with main campuses situated in the Klang Valley. Prior study approval was obtained from the Ethics Committee of each institution.

Our inclusion criteria were pregnant Malaysian women aged 18 and above, and able to understand Bahasa Malaysia. We excluded women with abnormal fetuses or stillbirths from this study. Participants were recruited among expectant mothers who received antenatal care as an outpatient and those who were admitted to the obstetric ward for delivery or other medical complications. The participants were selected through a convenient sampling method by the researchers. Eligible women were invited to complete a self-administered electronic questionnaire through a Google form, which included a consent section, that guaranteed complete individual anonymity during data analysis and research publication. Participation in this study was voluntary and none of the women received any gift or monetary compensation. Socio-demographic and clinical data were included in the data collection.

### 2.1. Instruments

The survey was conducted in the country's official language, Bahasa Malaysia. Eligible women were provided with a QR code link to the google form. Women who selected the option “I agree to participate” in the consent section, would be provided access to the questionnaire, while those who declined would not be able to proceed further and will be considered non-responders. We did not collect any demographic or clinical data from the non-responders.

We requested all participants to respond to each item in the google form before moving to the subsequent section; to minimize the risk of incomplete data. Those without internet access or who could not use their mobile phones would complete a paper-based questionnaire, and the responses would be transferred electronically by the investigator. The written consent forms of the paper-based respondents would be kept in their respective medical records. The questionnaire consists of several components:

#### 2.1.1. COVID-19 infection and vaccination status

Participants were asked about their history of COVID-19 infection and complications to themselves and their social contacts. We also collected data on (1) the individual's vaccination status, including the number of doses and type of vaccine, and (2) the source of vaccine information.

#### 2.1.2. Maternal perception and attitude toward COVID-19 vaccination and maternal vaccine acceptance

Women were asked about their perception and attitude toward COVID-19 vaccination using a thirteen-item questionnaire. The questionnaire was developed based on literature reviews and discussions among experts, including obstetricians and psychologists. Perception is defined as the way individuals interpret their experiences (19) while attitude refers to a set of emotions, beliefs, and behaviors toward a particular object, person, thing, or event (20).

The participants were asked to rate each statement using a 5-point Likert scale from 1 (strongly disagree) to 5 (strongly agree). Items 1 and 2 addressed the consent and information on vaccination. Items 3–6 covered the protective effect of the vaccine. Items 6–7 were related to maternal anxiety about the side effects of the vaccine on themselves and their babies. Item 8 assessed maternal worry about getting COVID-19 infection despite vaccination. Item 9 addressed maternal preference for post-partum vaccination. Items 10–11 assessed the maternal view on vaccination as a societal responsibility. Items 12–13 addressed vaccine choice and willingness to pay.

For statistical purposes, the individual responses were categorized as “Disagree” (those who responded disagree and strongly disagree i.e., Likert scale score 1–2), “Neutral” (neither agree nor disagree i.e., Likert scale 3), and “Agree” (those who answered agree or strongly agree i.e., Likert scale 4–5).

Maternal vaccine acceptance was calculated based on the number of women who responded “agree” or “strongly agree” (which corresponded to a score of 4 or 5 on the Likert scale) for Item 1 *(I agree to be vaccinated)*.

#### 2.1.3. COVID-19 pregnancy-related anxiety

This questionnaire consists of five items that evaluate maternal anxiety concerning COVID-19 infection to self and baby as well as pregnancy complications such as miscarriage, fetal anomaly, and preterm birth. This questionnaire demonstrated good internal consistency with Cronbach's alpha of 0.928 (21). The total score ranged between 5 to 25, and a 50% cut-off level (score ≥13) indicates greater maternal anxiety.

#### 2.1.4. General anxiety disorder

GAD-7 measures generalized anxiety disorder, social anxiety, panic disorder, and post-traumatic stress disorder. It consists of 7 items, each scored from 0 to 3, with total scores ranging from 0 to 21. The GAD-7 anxiety score is categorized into minimal (0–4), mild (5–9), moderate (10–14), and severe (15–21). The Bahasa Malaysia version of GAD-7 was validated in the primary care setting by Sidik et al. (22) and demonstrated good sensitivity, specificity, and concurrent and convergent validity (22). Women in our cohort who scored ten or above were considered to have a greater level of anxiety.

#### 2.1.5. Face validation

A face validation was conducted before the data collection in one of the centers; Universiti Kebangsaan Malaysia Medical Center. The questionnaire was distributed among twenty women (pregnant and non-pregnant) to assess its suitability. All women reported that the questionnaire was acceptable and easy to understand. The responses from the pregnant participants were not included in the final analysis.

### 2.2. Statistical analysis

Assuming that the vaccine acceptance rate among our cohort was 50%, The calculated sample size for this study was 384 participants, taking into account a 95% confidence interval, the limit of precision of 5%, with a design effect of 1.0 (23). The study data were analyzed using the Statistical Package of Social Sciences (SPSS) Version 26.0 (IBM Corp., Armonk, NY, USA). Continuous and categorical data were presented as mean (standard deviation, SD) or number, *n* (percentage, %), respectively.

A Chi-square test and univariate analysis were performed to determine the significant demographic factors associated with maternal vaccine acceptance. In addition, we evaluated the effect of maternal generalized anxiety and COVID-19 pregnancy-related anxiety on vaccine acceptance. All demographic variables with *p* < 0.10 in the univariate analysis were subsequently entered into the multivariate model. The following factors: maternal age and parity were also included in our model based on their significance in previously published studies (18, 24). The statistical analysis was conducted using Enter method and two-tailed *p*-value, to produce adjusted odd ratios (AORs) and the corresponding 95% confidence interval.

The reliability of our newly designed 13-item questionnaire was assessed using the Cronbach Alpha. Cronbach's alpha > 0.7 is regarded as satisfactory. The correlations between maternal vaccine acceptance, perception, and attitude toward COVID-19 vaccination were assessed using Spearman's correlation. The correlation scale is as follows; weak (<0.40), moderate (0.40–0.69), and strong ≥ 0.7) (25). We consider a result with a p*-*value < 0.05 as statistically significant.

## 3. Results

A total of 1,317 women were recruited with a 96.6% response rate, making the final number for analysis 1,272. The demographics and clinical characteristics are displayed in Table 1. The mean (SD) age for our cohort was 32.2 (4.6) years old, and the majority were Malays (89.5%). Over four-fifths of our respondents had tertiary education, and almost 80% were in employment. Approximately a quarter of our respondents or partners worked in the health sector. Eighty-three percent of our respondents were antenatal mothers with a mean (SD) gestation of 29.9 (7.7) weeks. A third of our cohort was nulliparous, and around 36% had medical or obstetric complications.

**Table 1:** Maternal demographics and clinical characteristics.

**Maternal characteristics**	***n*** **(%)**
Age, mean (SD)	32.2 (4.6)
**Ethnicity**, ***n*** **(%)**	
Malay Chinese Indian Others	1,139 (89.6) 73 (5.7) 32 (2.5) 28 (2.2)
**Education**, ***n*** **(%)**	
Primary Secondary Tertiary	2 (0.2) 214 (16.8) 1,056 (83.0)
**Employment**, ***n*** **(%)**	
Employed/self-employed Housewife	1,009 (79.3) 263 (20.7)
**Household income**, ***n*** **(%)**	
<RM 2000 RM 2000–4999 RM 5000–10000 >RM10000	140 (11.0) 567 (44.6) 459 (36.1) 106 (8.3)
Health sector employment, *n* (%)	312 (24.5)
**Parity, mean (SD)**	1.3 (1.27)
Nulliparous Multiparous	410 (32.2) 862 (67.8)
**Pregnancy status**, ***n*** **(%)**	
Antenatal Postnatal Post vaginal birth Post cesarean delivery	1,057 (83.1) 215 (16.9) 116 (54.0) 99 (46.0)
Gestation, mean (SD)	29.9 (7.7)
**Medical or obstetric complication**, ***n*** **(%)**	460 (36.2)
Hypertensive disorder Diabetes Anemia Others	58 (4.6) 202 (15.9) 117 (9.2) 121 (9.5)

Table 2 depicts the COVID-19 infection and the vaccination status among our cohort. Approximately 64% of the respondents or their social contact had COVID-19 infection. However, the majority of respondents who had COVID-19 infection underwent home quarantine, and just over a third required hospital admission. Almost 98% of respondents completed two doses of the COVID-19 vaccine while only one completely declined vaccination. The majority of respondents received the Pfizer vaccine (70%) followed by Sinovac (20%). The majority of our women obtained their vaccine information from the internet (69.2%) and social media such as Facebook and Instagram (53.7%). Around forty-five percent of respondents consulted health professionals on a matter concerning the COVID-19 vaccine.

**Table 2:** COVID-19 infection and vaccination status.

**Status**	***n*** **(%)**
**History of COVID-19 infection (self or contact)**	809 (63.6)
Self Household Family/relatives Other social contacts (friends/work colleagues)	288 (22.6) 232 (18.2) 440 (34.6) 340 (26.7)
**COVID-19 complications to family/social contact**	
ICU admission and Death	92 (7.2)
**COVID-19 to self**	
Home quarantine Hospital admission	167 (58.0) 108 (37.5) 13 (missing data)
**Vaccination status**	
Completed both doses Received one dose Registered, awaiting appointment Declined	1,244 (97.8) 18 (1.4) 9 (0.7) 1 (0.1)
**Vaccine received**	
Pfizer Sinovac Astra Zeneca Others	893 (70.2) 254 (20.0) 112 (8.8) 3 (0.2)
**Source of vaccine information**	
Internet Social Media (FB, Instagram, others) Television Social app (WA, Telegram, MKN) Healthcare Professionals Family/Relatives Friends Printed Media	880 (69.2) 683 (53.7) 643 (50.6) 626 (49.2) 570 (44.8) 321 (25.2) 255 (20.0) 254 (20.0)

Table 3 demonstrates the maternal perceptions of COVID-19 vaccination. The maternal acceptance rate for our cohort was 77.1%. Over two-thirds of women felt they received adequate information on the COVID-19 vaccine, and almost three-quarters of them agreed that vaccination prevents severe disease and reduces the risk of death. Approximately 40% of our respondents were anxious about the vaccine side effects to themselves and potential harm to their babies. As a result, less than a third of women preferred post-partum vaccination. Almost 80% of our cohort agreed that getting vaccinated is a societal responsibility and would recommend COVID-19 vaccination to their family and friends. Around 56% of them would like to be allowed to choose their vaccine, and <30% of women were willing to buy the COVID-19 vaccine.

**Table 3:** Maternal COVID-19 vaccine perceptions and attitudes.

**Perception**	**Responses**, ***n*** **(%)**		
	**Agree**	**Neutral**	**Disagree**
1. I agree to be vaccinated	981 (77.1)	197 (15.5)	94 (7.4)
2. I receive enough information on the vaccine	906 (71.2)	269(21.1)	97 (7.6)
3. The vaccine will prevent me from getting COVID-19	815 (64.1)	301 (23.7)	156 (12.3)
4. The vaccine will protect against severe disease	949 (74.6)	227 (17.8)	96 (7.5)
5. The vaccine will reduce the risk of death due to COVID-19	937 (73.7)	235 (18.5)	100 (7.9)
6. I worry about the side effect of the vaccine on myself	501 (39.4)	473 (37.2)	298 (23.4)
7. I worry about the vaccine I received causing harm to my baby/newborn	512 (40.3)	430 (33.8)	330 (25.9)
8. I still worry about getting infected even after I completed my vaccination	739 (58.1)	354 (27.8)	179 (14.1)
9. I prefer to be vaccinated after delivery	386 (30.3)	367 (28.9)	519 (40.8)
10. I would recommend my family and friends to get vaccinated	1,000(78.6)	178 (14.0)	94 (7.4)
11. Getting the vaccine is a societal responsibility	1,011(79.5)	178 (14.0)	83 (6.5)
12. Vaccine recipients should be allowed to choose their vaccine type	715 (56.2)	382 (30.3)	175 (13.8)
13. I am willing to pay to get the vaccine of my choice	379 (29.8)	483 (38.0)	410 (32.2)

The associations between maternal characteristics and COVID-19 vaccine acceptance are shown in Table 4. Our final multivariate model is based on the adjustment of the following factors: age, parity, household income, education, employment, and health worker. A greater household income, employment status, and health worker are independent predictors of maternal vaccine acceptance.

**Table 4:** Associations between maternal characteristics and COVID-19 vaccine acceptance.

**Maternal characteristics**	**Maternal vaccine acceptance**					
	***n*** (%)^*^	***p*** **-value**	**Odd ratio (95% CI)**	***p*** **-value**	**AOR (95% CI)**	***p*** **-value**
**Age**						
< 35	656 (76.2%)	0.252	Ref	*p =* 0.252	Ref	*p =* 0.668
≥ 35	325 (79.1%)		1.18 (0.89 – 1.57)		1.07 (0.78 – 1.46)	
**Ethnicity**						
Malay ethnicity	880 (77.3%)	0.731	1.08 (0.71−1.64)	*p =* 0.732	1.14 (0.74 – 1.76)	*p =* 0.549
Non-Malay	101 (75.9%)		Ref		Ref	
**Household income**						
< RM 5000	605 (71.4%)	< 0.001	Ref	*p < * 0.001	Ref	*p < * 0.001
>RM 5000	476 (84.2%)		2.14 (1.62 – 2.83)		1.87 (1.38 – 2.54)	
**Education**						
Non-tertiary education	149 (69.0%)	0.002	Ref	*p =* 0.002	Ref	*p =* 0.484
Tertiary education	832 (78.8%)		1.67 (1.21 – 2.31)		1.13 (0.80– 1.61)	
**Employment**						
Employed/self-employed	808 (80.1%)	< 0.001	2.09 (1.55-2.82)	*p < * 0.001	1.52 (1.10 – 2.10)	*p =* 0.011
Housewife/ unemployed	173 (65.8%)		Ref		Ref	
**Parity**						
Nulliparous	322 (78.5%)	0.408	Ref	*p =* 0.408	Ref	*p =* 0.339
Multiparous	659 (76.5%)		0.89 (0.67 – 1.18)		0.86 (0.64 – 1.17)	
**Medical condition**						
Yes	364 (79.1%)	0.199	1.20 (0.91 – 1.58)	*p =* 0.200	1.15 (0.86 – 1.52)	*p =* 0.346
No	617 (76.0%)		Ref		Ref	
**Health sector worker**						
Yes	267 (85.7%)	< 0.001	2.04 (1.44 – 2.89)	*p < * 0.001	1.79 (1.25- 2.57)	*p =* 0.001
No	714 (74.4%)		Ref		Ref	
**Internet and Social apps/media as an information source**						
Yes	552 (80.8%)	0.001	1.60 (1.13 – 2.25)	*p =* 0.007	1.63 (1.14 – 2.33)	*p =* 0.008
No	429 (72.8%)		Ref		Ref	
**COVID-19 infection to self or contact**						
Yes	648 (80.1%)	0.001	1.57 (1.20 – 2.05)	*p =* 0.001	1.33 (1.01 – 1.75)	*p =* 0.044
No	333(71.9%)		Ref		Ref	
**Maternal general anxiety**						
Yes	70 (78.7%)	0.722	1.10 (0.65 – 1.86)	*p =* 0.722	1.18 (0.69 – 2.02)	*p =* 0.540
No	911 (77.0%)		Ref		Ref	
**Greater COVID-19 pregnancy-related anxiety**						
Yes	932 (78.1%)	0.001	2.19 (1.36 – 3.51)	*p =* 0.001	2.42 (1.48 – 3.97)	*p < * 0.001
No	261 (21.9%)		Ref		Ref	

^*^Chi-square test; AOR, adjusted odd ratio; CI, confidence interval, Ref reference; AOR, adjusted for age, parity, household income, education, employment, and health worker.

Internet and social media use is associated with increased odds of accepting the COVID-19 vaccine among our cohort (*p* = 0.008). History of COVID-19 to self or among social contact is also a positive predictor for maternal willingness toward COVID-19 vaccination. Mothers who reported a greater COVID-19 pregnancy-related anxiety are twice more likely to accept COVID-19 vaccination, whilst generalized maternal anxiety does not seem to have an impact on maternal vaccine acceptance.

Our 13-item questionnaire demonstrated good internal consistency with a Cronbach's alpha value of 0.872. Table 5 demonstrates Spearman's correlations between various perceptions toward COVID-19 vaccination. There are strong positive correlations between vaccine acceptance and the perceptions of (1) receiving adequate information (0.785, *p* < 0.001), (2) protective effects of vaccination against the COVID-19 infection and its complications (0.692-0.828, *p* < 0.001), and (3) getting vaccinated as a societal responsibility (0.777, *p* < 0.001). Adequate information on vaccination is also significantly correlated with the favorable perceptions of the protective benefits of the COVID-19 vaccine against severe disease (0.733, *p* < 0.001) and death (0.703, *p* < 0.001). There is a significant correlation between the perception of vaccination as a societal responsibility and recommending vaccination to family and friends (0.909, *p* < 0.001). There are moderate correlations between the preference to receive postnatal vaccination and anxiety about the COVID-19 vaccine maternal (0.475, *p* < 0.001) and fetal side effects (0.514, *p* < 0.001).

**Table 5:** Spearman's correlation between maternal perceptions and attitudes toward COVID-19 vaccination.

**Maternal perception**	**Vaccine acceptance**	**AdequateInformation**	**COVID-19Protection**	**Prevents severe disease**	**Death prevention**	**Side effects**	**Harmful to baby**	**COVID-19 anxiety**	**PNvaccination**	**Vaccines for family & friends**	**Societal responsibility**	**Vaccinechoice**	**Vaccine purchase**
Vaccine Acceptance	1	0.785^**^	0.692^**^	0.828^**^	0.782^**^	0.059^*^	−0.020	0.285^**^	−0.188^**^	0.781^**^	0.777^**^	0.279^**^	0.230^**^
Adequate information		1	0.658^**^	0.733^**^	0.703^**^	0.111^**^	0.029	0.288^**^	−0.113^**^	0.667^**^	0.672^**^	0.286^**^	0.247^**^
COVID-19 Protection			1	0.762^**^	0.743^**^	0.097^*^	0.025	0.221^**^	−0.103^**^	0.611^**^	0.619^**^	0.265^**^	0.262^**^
Prevents severe disease				1	0.893^**^	0.104^**^	0.039	0.292^**^	−0.130^*^	0.734^**^	0.732^**^	0.302^**^	0.248^**^
Death prevention					1	0.118^**^	0.062^*^	0.295^**^	−0.122^**^	0.723^**^	0.715^**^	0.287^**^	0.245^**^
Vaccine side effects worry						1	0.769^**^	0.535^**^	0.475^**^	0.125^**^	0.124^**^	0.365^**^	0.156^**^
Harmful to baby							1	0.534^**^	0.514^**^	0.054	0.063^*^	0.355^**^	0.165^**^
COVID-19 anxiety								1	0.267^**^	0.347^**^	0.333^**^	0.352^**^	0.217^**^
Postnatal vaccination									1	−0.113^**^	−0.101^**^	0.315^**^	0.132^**^
Vaccines for family & friends										1	0.909^**^	0.340^**^	0.254^**^
Societal responsibility											1	0.366^**^	0.243^**^
Vaccine choice												1	0.354^**^
Vaccine purchase													1

Significance level:

* p < 0.005;

** p < 0.001.

## 4. Discussion

COVID-19 vaccine acceptance rate among the general population varies between 22 and 93%, with gender, age, education, and occupation as the significant socio-demographic determinants (26). Pregnant women demonstrate a lower level of acceptance of the COVID-19 vaccine, with rates ranging from 13.7 to 77% (26, 27). A study conducted in Japan during the first year of the pandemic demonstrated a high rate of vaccine hesitancy among pregnant women with primipara twice more likely to be vaccine-hesitant (odd ratio OR 2.38, *p* = 0.04) (28). The vaccine acceptance of our cohort of urban Malaysian mothers (77.1%) is among the highest reported in the literature.

Older mothers and higher education levels are associated with greater maternal willingness toward COVID-19 vaccination (29, 30). A systematic review by Nindrea et al. (31) found that pregnant women above 35 years were twice more likely to be receptive to the SARS-CoV-2 vaccine (pooled odd ratio, POR 2.01, 95%CI 1.10–2.93) (31). Older women are more likely to develop age-related chronic conditions such as diabetes and cardiovascular disease, which may increase their susceptibility to COVID-19-related morbidity and death, resulting in greater acceptance of vaccination (32). Our study showed that women aged above 35 reported a higher trend of vaccine acceptance, though the result was non-significant.

Previously published data showed a positive association between higher education and maternal vaccine acceptance (18, 31, 33, 34). A more educated individual may have easier access to vaccination facts and be able to interpret them better (31). Meanwhile, a lesser informed person is more likely to be affected by vaccine misinformation which may result in vaccine hesitancy. Our cohort reported increased odds of accepting the COVID-19 vaccine among individuals who received tertiary education; however, the factor was not significant in multivariable analysis.

We found that higher household income and employment were significant determinants of COVID-19 vaccine acceptance. Our study used the cut-off of RM 5000, based on the bottom forty percent (B40) definition of Malaysian household income by the Department of Statistics Malaysia (35). Women in our cohort with higher household income (>RM5000) were twice more likely to be receptive to the SARS-CoV-2 vaccine (*p* < 0.001). A survey conducted in sixteen countries during the pandemic confirmed the link between low education and low income with vaccine non-acceptance (16). An observational trial from the Global Network for Women and Children's Health Research involving seven low- and middle-income countries also confirmed that those with lower educational status were less willing to be vaccinated (36). Full-time employment likely indicates higher education and income, which explains the greater vaccine reception among employed women in our cohort. Similar to our study, Snazjder et al. (37) found that pregnant women who were employed full-time were twice likely more willing to receive the COVID-19 vaccine (AOR 2.22; 95% CI 1.02, 4.81) (37).

Being a health professional during the COVID-19 pandemic could carry an increased risk for COVID-19 infection due to greater exposure to the SARS-CoV-2 virus. A higher vaccine acceptance rate among healthcare workers than among non-healthcare workers or the general population (38, 39) may be explained by the perceived risk of contracting the infection through direct involvement with COVID-19 patients or a greater level of medical knowledge (39). Health sector employment among our respondents or their partner is also an independent predictor of maternal vaccine acceptance in our cohort. Interestingly, Battarbee et al. (18) found no significant difference in the willingness to be vaccinated among pregnant healthcare professionals and those who are employed in other sectors (18). Our study demonstrated that a history of COVID-19 infection to self or social contact is positively associated with willingness to accept the SARS-CoV-2 vaccine. A global survey conducted during the first year of the pandemic found an increased odd of vaccine acceptance among pregnant women who had lost a loved one to COVID-19 (OR 2.82, 95% CI 2.03–3.94) (16). One's willingness to be vaccinated may be increased by having a personal connection to COVID-19, often motivated by personal stories (40).

Our recently published data indicate that over four-fifths of our obstetric patients expressed worry about the risk of COVID-19 infection to themselves and their babies (21). A study among pregnant individuals in Singapore during the early wave of the pandemic demonstrated that women who associated COVID-19 infection with fetal anomalies and intrauterine fetal death had significantly higher anxiety scores (41). We found a significant increase in the likelihood of vaccine acceptance among mothers who reported greater COVID-19 pregnancy-related anxiety. Our result concurs with that of Kiefer et al. (33), which showed that maternal concern about contracting COVID-19 and its impact on self and pregnancy was associated with a lower odd of COVID-19 vaccine hesitancy (AOR 0.76; 95% CI 0.70–0.82) (33).

Concerns about vaccine safety (to self and fetus), vaccine effectiveness, and vaccine side effects are among the commonly perceived barriers to COVID-19 vaccination among pregnant women (18, 28, 42, 43) and the general population (44). A recent observational study conducted across ten countries in Asia, Africa, and South America found that a decline in the acceptance of the COVID-19 vaccine among the general population was significantly associated with the increased risks of vaccine side effects (44). Battarbee et al. (18) reported that 82% of expectant mothers who were unwilling to be vaccinated during pregnancy, cited vaccine safety as a major concern (18). Interestingly, a survey among female healthcare workers of reproductive age in the USA reported that pregnant participants were six times more likely to delay and twice as likely to decline COVID-19 vaccination (*p* < 0.05) compared to non-pregnant women. Townsel et al. (45) found that the highest rates of concern were about the safety and effectiveness of the vaccine (45). Similarly, a study by Wang et al. (46) found that pregnancy status had influenced around 44% of non-vaccinated healthcare workers not to receive the SARS-CoV-2 vaccine (46).

In this study, around two-fifths of our respondents expressed concern about the side effects of the COVID-19 vaccine to themselves and potential harm to the baby. Fortunately, these concerns did not seem to influence our cohort's overall maternal vaccine acceptance negatively. Our study found a strong correlation between maternal perception of the protective effects of the COVID-19 vaccine and their willingness to be vaccinated. Women who perceived the vaccine benefited both mother and baby were also less likely to express vaccine hesitancy (AOR 0.25; 95% CI 0.14–0.44) (33). A global survey by Skjefte observed significantly increased odds of maternal vaccine acceptance among those who were confident in the efficacy of the SARS-CoV-2 vaccine (OR 6.68; 95% CI 5.90–7.56) (16). Unsurprisingly, less than a third of our respondents preferred to receive post-partum vaccination, reflecting that a significant proportion of women still were concerned about the potential harm of taking it during pregnancy.

Our study revealed a strong correlation between maternal willingness to be vaccinated and receiving adequate vaccine information. A meta-analysis by Nindrea et al. (31) found that sufficient information on the SARS-CoV-2 vaccine was linked to pregnant women's desire to receive COVID-19 vaccination (POR 1.94, 95% CI 0.94–2.95) (31). Health professionals play a crucial advocative role, especially in relaying credible information to expectant mothers. Keifer et al. (33) demonstrated lower odds of COVID-19 vaccine hesitancy among pregnant women who reported discussing vaccination with their OB/GYN provider (AOR 0.40; 95% CI 0.25–0.62) (33). A similar finding was also observed by Desai et al. (47) that women who had previously discussed the COVID-19 vaccine with a physician were significantly more likely to receive the vaccine during pregnancy (45.8 vs 26.0%, *p* = 0.04) (47).

Vaccine acceptance may be associated with various factors that differ across cultures, contexts, and settings. For example, in more collective cultures, concern for others and the perception of increased social acceptance may be an essential variable in the willingness to accept COVID-19 vaccination, as shown in Asian countries like China and Japan (48, 49). Fu et al. (48) concluded that appealing to a communal responsibility to protect others *via* indirect protection is one of the strategies to maintain COVID-19 vaccine uptake (48). Our cohort also reported a similar finding in which a strong positive correlation was observed between vaccine acceptance and the notion of vaccination effort as a societal responsibility. These women were also more likely to recommend the SARS-CoV-2 vaccine to their relatives and friends.

Healthcare providers play a pivotal role as reliable sources of vaccine information with a positive impact on maternal vaccine attitudes and uptake. Around 45% of our women referred to health professionals for vaccine advice. A survey among pregnant women from low- and middle-income countries reported that health professionals were among the most trusted sources of information for vaccination (36). Published data also demonstrated an increased likelihood of receiving vaccines among expectant mothers who had previously discussed COVID-19 with their physicians (30, 31, 47). An Italian study reported that expectant mothers whose main source of vaccine information was their gynecologists were almost three times more likely to receive the COVID-19 vaccine (AOR 2.92; 95% CI 1.58–5.42) (30).

Responsible dissemination of vaccine information is also essential in increasing vaccine uptake in the population. We found that the use of the internet, alongside social media and applications, has a positive influence on maternal vaccine acceptance among pregnant Malaysian women. The internet has been increasingly relied upon as a source of information, especially during the nationwide lockdown. Our local survey before the pandemic indicated that half of the pregnant women obtained their vaccine information from the internet, but the figure increased to around 70% during the COVID-19 outbreak (50). In contrast to traditional media, social media allow individuals to rapidly create and share content globally without editorial oversight. Unfortunately, false and misleading information about COVID-19, potentially dangerous treatments, and eventual vaccination continue to grow on social media platforms (13). As a previous study had demonstrated a direct correlation between exposure to misinformation and vaccine hesitancy (51); it is imperative that accurate and reliable “infodemic” is received by the general population.

### 4.1. Strengths and limitations

To our knowledge, our study is the first to assess the COVID-19 vaccine acceptance among pregnant women in Malaysia. The multi-centric design allowed us to cover a wider population across the Klang Valley and obtain a large sample, making the results more reliable and accurate.

Our cohort consists of urban women with a relatively high socio-economic status, which may account for the high level of maternal vaccine acceptance. Most of these women would have easy access to technology and vaccine information, contributing to a more positive view of the SARS-CoV-2 vaccine. A high level of knowledge of COVID-19 and good practice among these urban women (52), would have also contributed to the maternal willingness toward COVID-19 vaccination; in keeping with other published data (31, 53).

Our study has several limitations. The cross-sectional design of our research restricts the assessment of maternal vaccine acceptance to a specific point in time. A study by Germann et al. (54) demonstrated that vaccine hesitancy can be dynamic for each individual through the peripartum period (54). Rapidly evolving data on COVID-19 alongside the transition from pandemic to endemic phase will inevitably influence maternal attitudes toward vaccination. A longitudinal study would allow further understanding of this matter.

Although our survey had a high response rate, no data was available on the non-responders. There could be a non-response bias as we were unable to compare their characteristics to that of the participating women. There are a few contributory factors to the selective bias in our study that limit the generalizability of our findings. The convenient sampling in our study meant that our cohort was limited to women who attended teaching hospitals for obstetric care. The services provided by these teaching hospitals are subjected to medical fees, while the healthcare delivered by the public hospitals under the Ministry of Health (MOH), is normally free of charge to Malaysians. As our research did not include participants from government hospitals, we might have missed women with different demographic backgrounds. We also did not include women who attended local antenatal clinics run by the MOH or those who received antenatal care in the private sector.

The proportion of Malay women in our cohort is high (89.6%) in comparison to the general population at 57.8% (55). Our previous study demonstrated that mothers of Malay ethnicity reported a more positive perception of the Malaysian Control Order and a better obstetric care experience during the COVID-19 pandemic. These findings were attributed to the good underlying knowledge of COVID-19 and high regard toward health professionals and authority among Malay women (52). This cultural difference may influence the overall maternal vaccine acceptance in our cohort. We also did not recruit women from rural areas who may have difficulty accessing healthcare. Women who face barriers to accessing healthcare services were more likely to report COVID-19 vaccine hesitancy (26). Our study therefore may not reflect the true level of vaccine hesitancy among Malaysian expectant mothers nationwide. Hence, future research should include non-urban subjects as well as represent the true proportion of the Malaysian multi-ethnic population.

## 5. Conclusion

The high level of COVID-19 vaccine acceptance observed among Malaysian pregnant women is mainly attributed to their high socio-economic status. Willingness toward the SARS-CoV-2 vaccine is strongly influenced by sufficient vaccine information and maternal belief in its protective benefits, alongside the notion of herd immunity as a communal effort. Health professionals and social media also play a pivotal role in disseminating accurate information on the COVID-19 vaccines to expectant mothers as part of the continuous drive to eliminate COVID-19. These important elements should be considered in planning strategies to improve maternal vaccine uptake and will help the policymaker in future vaccination programs.

## Data availability statement

The original contributions presented in the study are included in the article/supplementary material, further inquiries can be directed to the corresponding author.

## Ethics statement

The studies involving human participants were reviewed and approved by the Research Ethics Committee of the National University of Malaysia. The patients/participants provided their written informed consent to participate in this study.

## Author contributions

Conceptualization: AK, SS, and IKA. Methodology: AK and RAR. Formal analysis: AK and SS. Investigation: AK, WRD, BA, MK, and RAD. Writing—original draft preparation: AK and WRD. Writing—review and editing: BA, IKA, and RAR. Project administration: AK. All authors contributed to the article and approves the submitted version.
